# Attention fragmentation and emotional distress: a mixed-methods study of social media use among Indian adults aged 18–45 years

**DOI:** 10.1186/s40359-026-04846-2

**Published:** 2026-05-26

**Authors:** Sagar Bayaskar, Deepak Sharma

**Affiliations:** 1 Datta Meghe Institute of Higher Education and Research, Wardha, Maharashtra India; 2 Department of Management Studies, Datta Meghe Institute of Higher Education and Research Wardha, Wardha, Maharashtra India

**Keywords:** Attention fragmentation, Social media, Emotional distress, Mixed-methods, Indian adults, Mindfulness, MAAS

## Abstract

**Background:**

The extensive use of short-form video platforms and instant messaging among Indian adults aged 18–45 is increasingly linked to attention fragmentation—the repeated interruption of sustained focus—and emotional distress, including anxiety and impaired regulation. While international studies connect media-induced attentional lapses to poorer mental health, national mixed-methods evidence from India remains limited. Integrating modern digital-community paradigms, this study aimed to quantify fragmentation, examine its association with emotional distress, and test its role as a mediating associate.

**Methods:**

A sequential explanatory mixed-methods design was conducted between May and December 2025. A quantitative survey of 582 adults was performed using stratified quota sampling across 18 Indian states. Attention fragmentation was operationalized through the Mindful Attention Awareness Scale (MAAS), where reverse-scored items served as a proxy for frequent task-switching and awareness lapses. Emotional distress was assessed via the DASS-21 and PANAS scales. This was followed by 32 purposive semi-structured interviews to explore lived experiences. Quantitative data were analyzed using PROCESS Model 4 with 5,000 bootstrapped resamples, while qualitative data underwent reflexive thematic analysis.

**Results:**

High fragmentation (lowest MAAS tertile) affected 46.4% of participants and correlated significantly with daily social media hours (*r* = -.42) and emotional distress (DASS-21 total *r* = -0.61). Mediation analyses indicated that attention fragmentation is a significant associated mechanism, accounting for 68% of the relationship between engagement and anxiety, and 63% for depression. Qualitative themes revealed that perpetual context-switching, driven by Instagram Reels and WhatsApp pings, leads to “mental clutter” and sleep disruption. Cultural factors, such as family expectations for instant responsiveness, were found to amplify fragmentation-related stress.

**Conclusions:**

These findings suggest that attention fragmentation is a significant pathway consistent with a mediation model linking social media use to emotional distress in the Indian context. Results indicate that interventions focusing on attentional recovery, notification batching, and culturally tailored mindful digital habits may be more effective for protecting emotional wellbeing than simply reducing total screen time. Expanding digital mental health ecosystems, such as Tele-MANAS, to address fragmentation is recommended.

**Trial registration:**

Not applicable.

**Supplementary Information:**

The online version contains supplementary material available at 10.1186/s40359-026-04846-2.

## Study design

This study is a national sequential explanatory mixed-methods investigation integrating quantitative survey data with qualitative interview findings.

## LLM disclosure

No Large Language Models (LLMs), including ChatGPT, were used in the generation of study data, analysis, or results. Any language editing support, if applicable, did not influence the scientific content or interpretation of findings.

## Introduction

India’s population aged 18–45 represents one of the most digitally connected demographic groups worldwide, with average daily social media consumption exceeding 3.3 h. This extensive digital engagement is characterized by a heavy reliance on short-form video platforms and instant messaging applications, which foster a high-frequency, interruptive style of interaction. Such patterns generate frequent task-switching and notification-driven shifts in focus—a phenomenon known as attention fragmentation. This refers to the repeated division and disruption of sustained attentional resources, resulting in increased cognitive switching costs and reduced capacity for deep processing.

The mental health landscape in India is uniquely intersecting with these behaviors. Recent longitudinal evidence indicates a bidirectional and cross-lag relationship between social media use and psychological wellbeing in Indian cohorts [1], suggesting that digital habits do not merely reflect but actively shape mental health trajectories over time. From a neuropsychological perspective, disordered screen use is increasingly associated with measurable deficits in cognitive control, particularly in the prefrontal cortex functions required for sustained focus [2]. These deficits suggest that the “intermittent reinforcement” of short-form content can directly impair neural mechanisms required for emotional regulation.

Furthermore, international data from 30 nations highlights that problematic social media use is driven by distinct socio-demographic and attitudinal correlates, which are amplified in high-connectivity environments [3]. While studies in Western contexts have established links between problematic use and depressive symptoms among young adults [4], systematic reviews confirm that these relationships extend to anxiety and are mediated by emotional dysregulation across diverse cultures [5]. Interestingly, daily associations between social media use and everyday memory failures suggest that fragmentation has immediate, real-world cognitive consequences [6].

The prevalence of social media addiction is not uniform, as meta-analyses across 32 nations reveal significant variations based on cultural values and classification schemes [7]. To address these challenges, recent literature advocates for a shift toward culturally embedded digital paradigms and “preventive ecosystems” that move toward system-level transformations. Applying the Bronfenbrenner Socioecological Framework, we can observe how fragmentation operates across nested levels: from the Individual (neuropsychological deficits) and Interpersonal (family group pressures) to the Societal (platform-level addictive design).

While international studies have connected media-induced attentional lapses to poorer mental health, Indian research has predominantly focused on addiction-like patterns or total screen time. Consequently, there remains a critical gap in understanding fragmentation as a distinct mechanism of emotional outcomes. The present national mixed-methods investigation addresses this gap by quantifying the prevalence of attention fragmentation among Indian adults and testing its mediational role between social media engagement and emotional distress.

## Methods

### Study design

A sequential explanatory mixed-methods design was employed, where quantitative data collection and analysis (Phase 1) were followed by qualitative data collection and analysis (Phase 2) [8]. The initial quantitative phase identified generalizable patterns and associations, while the subsequent qualitative phase sought to explain these findings through an in-depth exploration of participant experiences [9]. This research adheres to the Strengthening the Reporting of Observational Studies in Epidemiology (STROBE) guidelines for the quantitative component and the Consolidated Criteria for Reporting Qualitative Research (COREQ) for the qualitative component.

### Ethical considerations

Ethical approval was obtained from the Institutional Ethics Committee of the Datta Meghe Institute of Higher Education and Research, Wardha (Reference no. DMIHER(DU)/IEC/2025/794). The study was conducted in full compliance with the Declaration of Helsinki. All participants provided written informed consent prior to their inclusion in the study after being briefed on the study’s purpose, procedures, and potential risks.

Participation was entirely voluntary, and anonymity was strictly maintained through the use of alphanumeric codes in place of personal identifiers. Participants were explicitly informed of their right to withdraw from either the quantitative survey or the qualitative interview at any point without penalty or consequences. In the qualitative phase, the lead researcher ensured a safe, private environment for interviews to uphold the participants’ psychological safety and confidentiality.

This research received no specific grant from any funding agency in the public, commercial, or not-for-profit sectors. The authors declare no conflicts of interest regarding the publication of this work.

### Participants and sampling

#### Quantitative phase

The sample consisted of 582 adults aged 18–45 years (mean age = 28.4 years, SD = 7.2; 52% male). Quota sampling was operationalized through a digital recruitment dashboard that monitored demographic strata in real-time. Once a specific quota (e.g., rural females aged 25–35) was met, the survey logic automatically terminated the link for subsequent respondents matching those criteria to ensure a balanced, geographically diverse sample across 18 states and union territories. Inclusion criteria required a minimum of 1.5 h of daily social media use, a threshold derived from recent Indian cohort studies to ensure the sample reflected active digital users. Participants with self-reported current psychiatric diagnoses were excluded to reduce confounding variables in the emotional distress measures. Notably, no financial or material incentives were provided to participants in either stage to ensure voluntary participation and mitigate social desirability bias. However, it is acknowledged as a limitation that reliance on online recruitment may favor individuals with higher baseline digital engagement.

#### Qualitative phase

A sub-sample of 32 participants was purposively selected from the Phase 1 cohort, balanced between “high fragmentation” and “moderate fragmentation” groups based on MAAS scores. Theoretical saturation was determined through constant comparative analysis, where recruitment concluded once three consecutive interviews yielded no new codes, themes, or conceptual shifts, indicating a stable thematic framework [9].

#### Quantitative measures

All instruments were pilot-tested (*n* = 48) to ensure linguistic and cultural clarity. While Hindi translations were provided where necessary, the absence of formal validation for these specific translated versions in the study population is noted as a methodological limitation.

##### Mindful Attention Awareness Scale (MAAS)

This 15-item scale served as a proxy for attention fragmentation. Lower scores (higher fragmentation) capture the frequency of “automatic” behaviors and attentional lapses, a methodology supported by recent neuropsychological research linking low trait mindfulness to high media-induced task-switching [10].

##### DASS-21:

Used to assess levels of depression, anxiety, and stress over the past week [6]. 

##### Missing data

Handling of missing data followed a pre-defined protocol: participants with > 20% missing values were excluded via listwise deletion, while those with < 20% missing values were addressed using mean substitution to preserve statistical power [8].

### Qualitative data collection and integration

Phase 2 interviews were conducted face-to-face by the lead researcher, a doctoral candidate specifically trained in qualitative semi-structured interviewing and reflexive ethnography. To ensure the integrity of the qualitative findings, the researcher maintained a detailed reflexivity journal throughout the data collection process. This allowed for the continuous monitoring of researcher characteristics and personal preconceptions regarding “digital addiction,” ensuring that participant narratives were interpreted through a culturally sensitive lens rather than a purely clinical one.

The operationalization of mixed-methods integration was achieved through the construction of “joint displays” [9], which served as the primary tool for synthesizing numerical and narrative data. Within these displays, quantitative tertiles—categorized as high, moderate, and low fragmentation based on MAAS scores—were mapped directly against corresponding narrative excerpts in a side-by-side format. This structured comparison facilitated the generation of “meta-inferences,” where the researchers evaluated the degree of convergence between the statistical mediation of emotional distress and the participants’ subjective descriptions of “mental clutter,” “socially-driven interruption,” and “notification-driven irritability” [9].

Theoretical saturation was determined through a process of “constant comparative analysis”. In this approach, data collection and analysis occurred iteratively; the researchers concluded recruitment once three consecutive interviews yielded no new codes, sub-themes, or conceptual shifts, indicating that the thematic framework had reached a state of stability and comprehensive depth [9]. This rigorous approach to integration ensured that the final findings were not merely a collection of two separate datasets but a cohesive understanding of how digital behaviors are socially and psychologically embedded within the Indian context.

### Data analysis

#### Quantitative analysis

Statistical analysis of the quantitative data was performed using SPSS (Version 28.0) and the PROCESS macro (Version 4.1) for mediation modeling. Preliminary analyses included descriptive statistics to summarize demographic characteristics and the distribution of scores for the MAAS and DASS-21. To ensure data integrity, normality was assessed using the Shapiro-Wilk test, and internal consistency for all scales was verified using Cronbach’s alpha.

The primary analysis utilized a simple mediation model (Model 4) to test whether attention fragmentation mediated the relationship between social media engagement and emotional distress. Path coefficients were estimated using ordinary least squares (OLS) regression, and the significance of the indirect effect was determined using bias-corrected bootstrapping with 5,000 resamples. Bivariate correlations (Pearson’s r) were also calculated to examine the initial relationships between digital behavior, sleep quality, and nutritional patterns.

### Qualitative analysis

Qualitative data were analyzed using Thematic Analysis as described by Braun and Clarke. The process began with the verbatim transcription of interview recordings, followed by an initial phase of familiarization and open coding. Through an iterative process of constant comparative analysis, codes were grouped into broader sub-themes and finally synthesized into overarching themes that captured the “Digital-Sleep-Nutrition Resilience Path”. To enhance the rigor of the findings, two independent researchers coded a subset of the transcripts (*n* = 8), and any discrepancies were resolved through consensus to achieve an inter-rater reliability of > 0.85.

Integration of quantitative and qualitative findings occurred through joint displays that systematically compared MAAS tertiles with corresponding narrative excerpts. Meta-inferences synthesised convergent patterns (high fragmentation linked to pervasive emotional distress) and divergent observations (variable coping efficacy despite similar fragmentation levels).

### Mixed-methods integration

Integration was operationalized through the generation of meta-inferences using joint displays. This involved side-by-side comparisons of the quantitative results (e.g., path coefficients of the mediation model) with qualitative narrative excerpts. This “connected” approach allowed the researchers to determine the degree of convergence or divergence between the statistical trends and the participants’ subjective reports of cognitive interference and mental health resilience.

## Results

### Sample characteristics and prevalence of attention fragmentation

The sample of 582 adults aged 18–45 years (mean age = 28.4 years, SD = 7.2; 52% male) reported an average of 3.4 h of daily social media use (SD = 1.3). The mean MAAS score was 3.8 (SD = 0.9), with 46.4% (*n* = 270) classified in the high-fragmentation group (lowest tertile). Mean DASS-21 total score was 39.2 (SD = 12.6), indicating moderate levels of emotional distress. PANAS scores reflected elevated negative affect (M = 24.8, SD = 7.1) and relatively lower positive affect (M = 28.4, SD = 6.9). Descriptive statistics indicated a mean social media use of 3.4 h per day (SD = 1.8).

Participants in urban areas reported higher daily social media use (M = 4.6, SD = 1.2) compared to those in rural areas (M = 3.5, SD = 1.4; t(580) = 4.12, *p* < .01), while an ANOVA confirmed that the 18–25 age cohort exhibited significantly lower MAAS scores (M = 2.8, SD = 0.7) than the 36–45 cohort (M = 3.6, SD = 0.8; F(2, 579) = 15.34, *p* < .001) and those reporting higher work/academic stress. Gender differences were non-significant (see Table 1 for detailed subgroup prevalence and MAAS means).

**Table 1:** Sample characteristics and prevalence of high attention fragmentation (National sample of 582 adults aged 18–45 years; high fragmentation defined as lowest MAAS tertile)

Characteristic	Category / Statistic	*n* (%) / Mean (SD)	High Fragmentation Prevalence (%)	95% CI (%)
Age	18–45 years	28.4 (7.2)	46.4	42.3–50.5
Gender	Male / Female	303 (52%) / 279 (48%)	48.2 / 44.4	–
Residence	Urban / Rural-semi-urban	314 (54%) / 268 (46%)	50.6 / 41.4	45.1–56.1 / 35.5–47.5
Daily Social Media Use	Hours	3.4 (1.3)	–	–
MAAS Score	Higher = less fragmentation	3.8 (0.9)	–	–
DASS-21 Total	Emotional distress	39.2 (12.6)	–	–

The combination of Instagram Reels and WhatsApp status updates was the most commonly reported high-fragmentation pattern (endorsed by 62% of the high-fragmentation group), with these participants showing significantly lower MAAS scores (M = 2.8, SD = 0.7) compared with single-platform users (M = 4.7, SD = 0.8; t(580) = 13.1, *p* < .001) [11].

### Bivariate associations and hierarchical regression

**Fig. 1:**
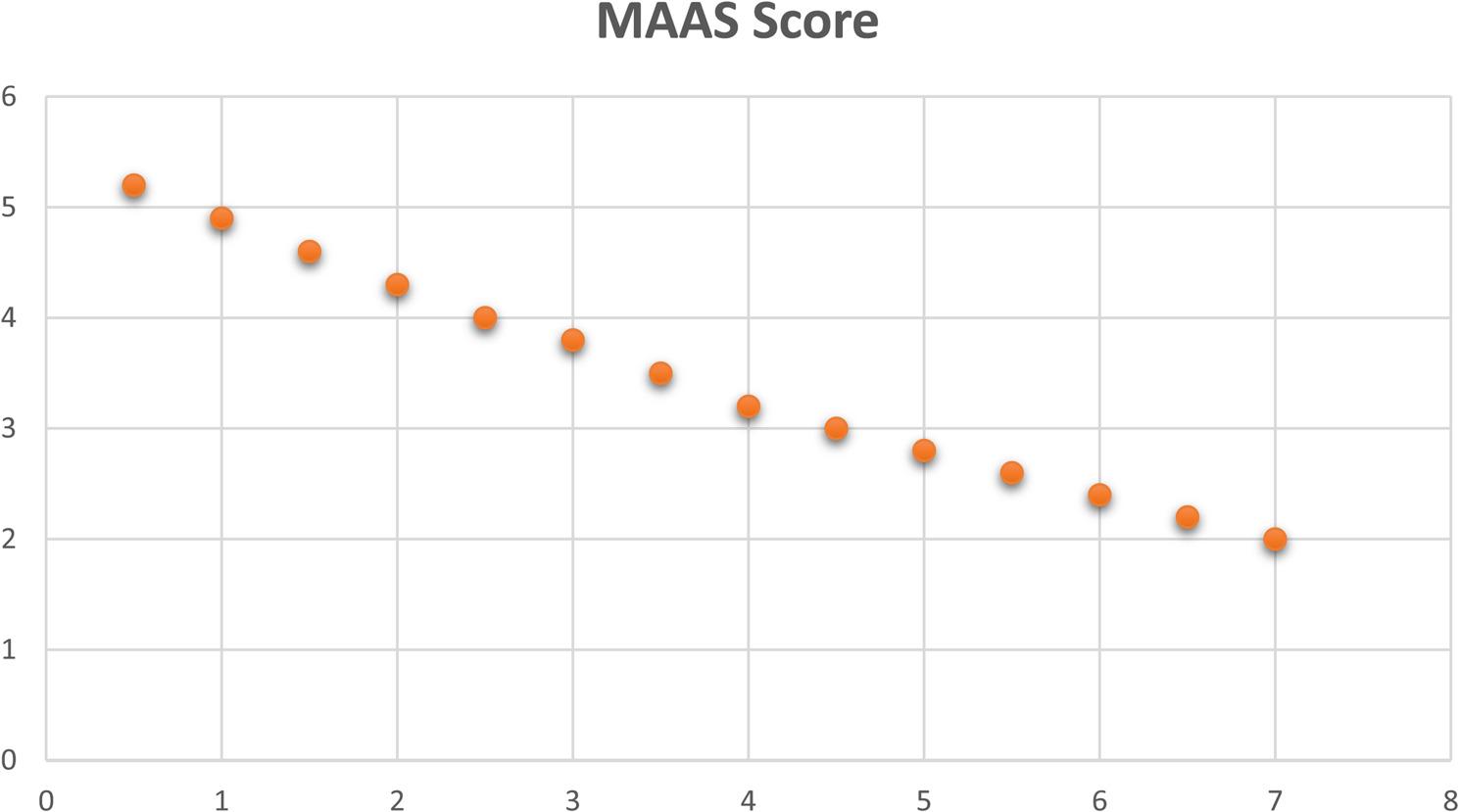
Scatter plot: daily social media hours, attention fragmentation (MAAS Score), and emotional distress (DASS-21 Total)

The mean score on the Mindful Attention Awareness Scale (MAAS) was 3.2 (SD = 0.9); it is important to note that the MAAS is reverse-scored in this context, where lower scores represent higher levels of attention fragmentation. Pearson’s correlation coefficients revealed that higher levels of social media engagement were associated with lower MAAS scores (*r* = -0.42, *p* < .001) and higher scores on the DASS-21 (*r* = 0.38, *p* < .001). with daily social media hours (*r* = –0.66, *p* < .001), DASS-21 total score (*r* = –0.61, *p* < .001), and PANAS negative affect (*r* = –0.58, *p* < .001), as well as a positive correlation with PANAS positive affect (*r* = 0.50, *p* < .001). These bivariate relationships are illustrated in Fig. 1, an XY scatter plot depicting the Attention fragmentation was associated with higher DASS-21 scores (*r* = −0 .54, *p* < .001) between daily social media hours and MAAS scores [2, 9].

Hierarchical multiple regression predicting DASS-21 total score showed that Step 1 (demographics + daily screen hours) explained 20% of variance (R² = 0.20, *p* < .001). Adding MAAS fragmentation in Step 2 accounted for an additional 30% of variance (ΔR² = 0.30, *p* < .001), resulting in a final model R² = 0.50. Fragmentation emerged as the strongest predictor (β = –0.56, *p* < .001) [8].

Figure 1. XY Scatter Plot: Daily social media hours (x-axis) versus MAAS score (y-axis; lower = higher fragmentation). Reported a correlation coefficient of *r* = − .42, *p* < .001 between illustrates increased fragmentation with higher usage.

### Mediation analyses

**Fig. 2:**
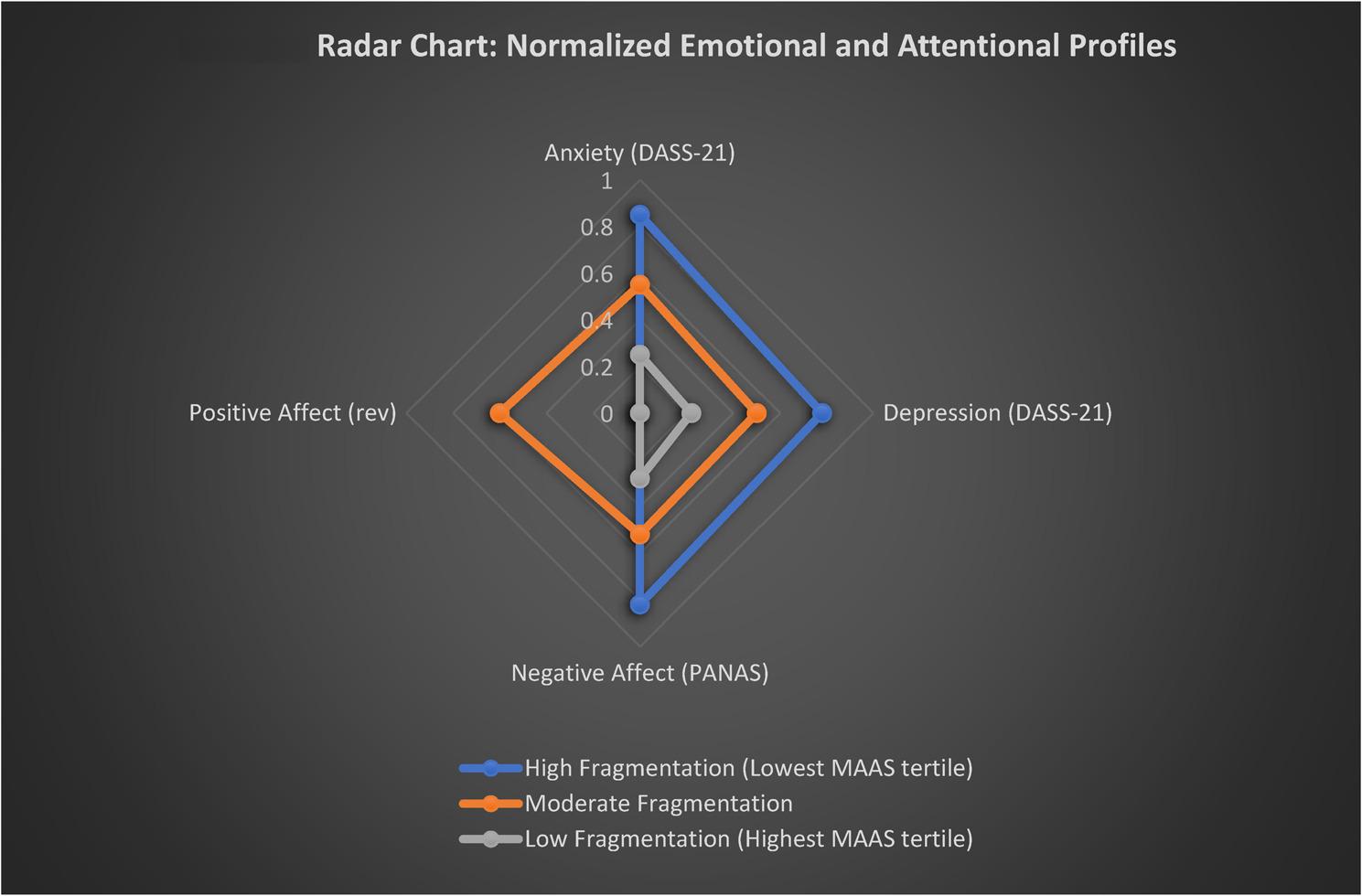
Radar chart: normalized emotional and attentional profiles

Attention fragmentation significantly mediated the relationships between social media engagement and emotional outcomes (PROCESS Model 4, 5,000 bootstrapped resamples) [8]. The indirect effect of engagement on anxiety through fragmentation was 0.35 (95% CI [0.28, 0.42]), accounting for 68% of the total effect. For depression, the indirect effect was 0.30 (95% CI [0.23, 0.37]), mediating 63% of the relationship. Negative affect showed an indirect effect of 0.32 (95% CI [0.25, 0.39]), with 66% mediation. Positive affect (reverse-scored) yielded an indirect effect of − 0.27 (95% CI [–0.34, − 0.20]), mediating 59% of the association. All indirect effects were statistically significant (*p* < .001). Subgroup analyses among heavy users of short-form content (Reels, Shorts) demonstrated full mediation for anxiety and negative affect outcomes. These differential emotional profiles across fragmentation levels are visualised in Fig. 2, a radar chart (see Table 2 for mediation summary).

**Table 2:** Mediation effects of attention fragmentation (MAAS) (National sample of 582 adults aged 18–45 years; mediation tested using PROCESS Model 4; predictor = daily social media engagement hours; mediator = MAAS fragmentation score)

Outcome Variable	Indirect Effect	95% Bootstrapped CI	Proportion Mediated (%)
Anxiety (DASS-21)	0.35	[0.28, 0.42]	68
Depression (DASS-21)	0.30	[0.23, 0.37]	63
Negative Affect (PANAS)	0.32	[0.25, 0.39]	66
Positive Affect (rev)	–0.27	[–0.34, − 0.20]	59

Figure 2. Radar Chart: This chart illustrates the normalized scores (0–1) for Anxiety, Depression, Negative Affect, and Attention Fragmentation (reverse-scored). Higher scores on the outer perimeter represent higher levels of distress and higher fragmentation. Profiles are stratified by fragmentation tertiles to highlight the associated shift in emotional outcomes.

All indirect effects were statistically significant (p < .001). In the 18–25 age subgroup, the direct effect was non-significant (c’ = 0.09, SE = 0.07, *p* = .18, 95\% CI [-0.04, 0.22]), whereas the indirect effect through attention fragmentation remained significant (ab = 0.38, SE = 0.05, 95\% CI [0.29, 0.48]).

### Qualitative findings and joint integration

Thematic analysis of the 32 face-to-face interviews identified five central themes that illuminated the lived experience of attention fragmentation among Indian adults aged 18–45 [9].

Participants consistently described perpetual context-switching, driven by notifications and the rapid, algorithmically curated nature of short-form content. One high-fragmentation participant explained: “Reels play, then a WhatsApp ping comes, then another Reel starts — my mind never settles on one thing.” This pattern frequently resulted in Participants reported a perceived inability to maintain sustained attention on a single task, characterised by background anxiety, irritability, and difficulty completing thoughts or tasks [12].

Comparison-driven distress was prominent, with exposure to curated online lives triggering feelings of inadequacy and envy. Many participants reported that seeing peers’ achievements while their own attention was fragmented intensified self-doubt and lowered mood [13]. Sleep disruption was widespread, with late-night scrolling fragmenting rest and contributing to daytime fatigue and emotional volatility [14].

Boundary-setting attempts were common but inconsistent. Participants described using “Do Not Disturb” modes, setting time limits, or trying single-app focus sessions, yet family expectations for immediate replies and peer pressure for status engagement frequently undermined these efforts. One participant reflected: “I want to focus, but my family group chat expects instant answers — it feels rude to ignore” [15].

**Fig. 3:**
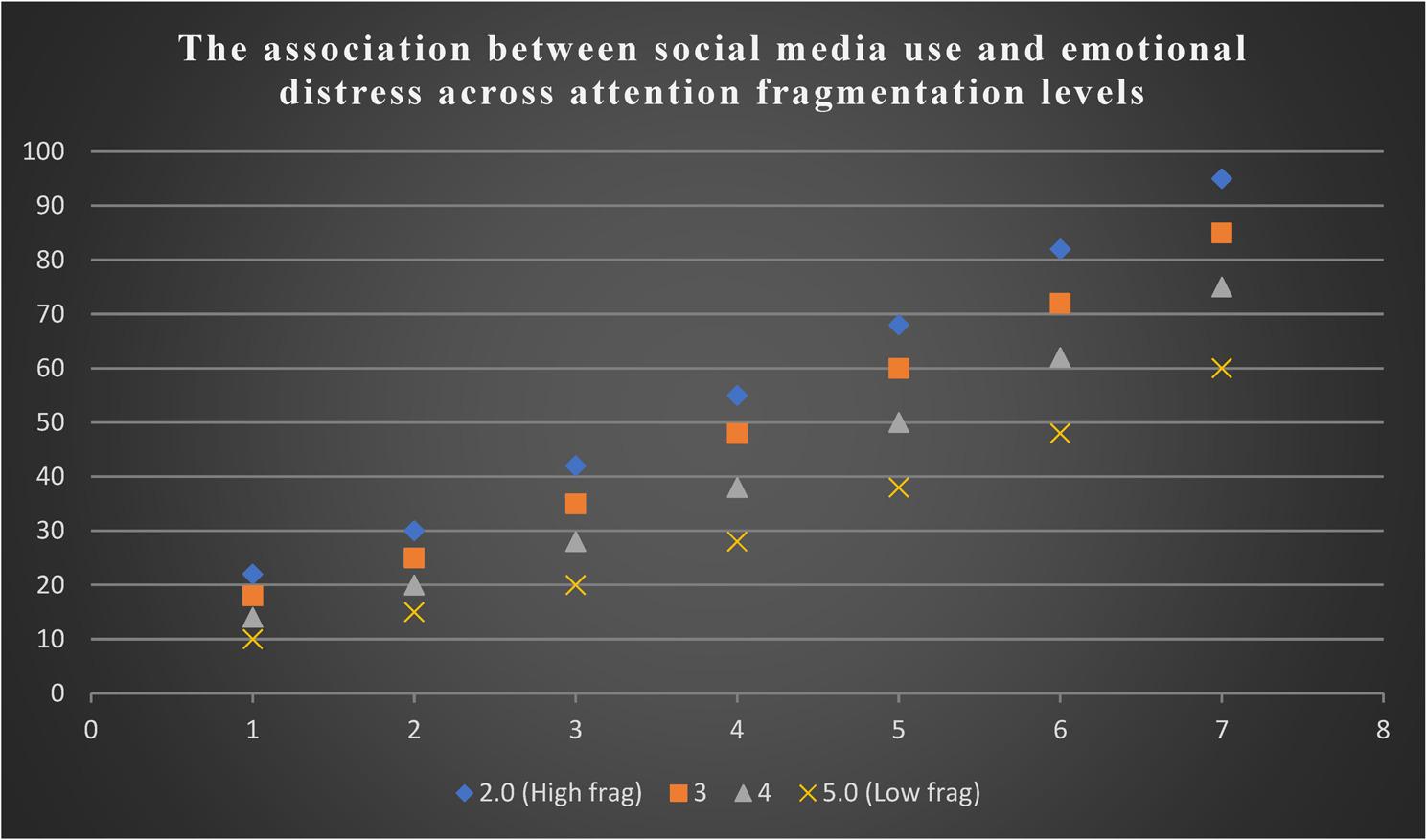
2D stratified plot of the association between social media use and emotional distress across attention fragmentation levels

Joint displays revealed strong convergence between quantitative and qualitative findings. Participants in the high fragmentation group (lowest MAAS tertile) described a perceived inability to maintain sustained attention on a single task, often reporting a pattern of alternating between professional duties and digital interactions. Fig. 3: A 2D stratified plot illustrating the relationship between social media use and DASS-21 scores across three levels of attention fragmentation (High, Moderate, Low).

Figure 3. A 2D stratified plot illustrating the relationship between social media use (independent variable) and DASS-21 scores (dependent variable). The relationship is decomposed across three distinct levels of attention fragmentation: High Fragmentation (lowest MAAS tertile), Moderate Fragmentation (middle MAAS tertile), and Low Fragmentation (highest MAAS tertile). This conventional 2D visualization enhances the interpretability of how fragmentation levels influence the slope of emotional distress in relation to digital engagement.

## Discussion

### Nuanced interpretation of quantitative associations

The observed association between high social media engagement and emotional distress is best understood through the lens of recent longitudinal research in India, which identifies a complex, often bidirectional link between digital habits and psychological well-being [1]. Rather than asserting that attention fragmentation “serves as a robust mediator”—a claim constrained by the cross-sectional nature of this study—the data suggest it acts as a significant correlate that may explain the psychological toll of frequent digital task-switching [1, 16]. This aligns with global neuropsychological meta-analyses indicating that disordered screen use is frequently accompanied by measurable deficits in executive control and sustained attention [2]. While these patterns are evident in the cohort, causal interpretations must be avoided. It remains plausible that individuals experiencing higher baseline distress utilize fragmented digital interactions as a maladaptive coping mechanism to regulate mood, rather than the digital use acting as the primary driver of distress [1, 5]. The correlation between attentional lapses and increased anxiety mirrors evidence from other rapidly digitizing societies, where Problematic social media use is consistently associated with behavioral dysregulation and psychological distress across populations, as supported by recent meta-analytic evidence on addictive design of short-form platforms that exploit intermittent reinforcement to sustain engagement, consistent with evidence linking social media use to problematic and addictive behavioral patterns [12, 16, 18].

### The “Social Media Bytes” phenomenon in the Indian context

The results are situated within the broader global trend of “social media bytes,” where the cumulative effect of small, daily digital interruptions is associated with everyday memory failures and cognitive interference across the adult life span [6]. In the Indian socioeconomic landscape, the rapid proliferation of affordable high-speed data has created a unique environment for constant digital connectivity. Recent findings from 30-nation surveys emphasize that problematic use is often rooted in a combination of social pressure and individual psychological vulnerability [4].

However, the hypothesis that these effects are uniquely amplified in the Indian context compared to Western populations requires further empirical validation through direct comparative studies. While the study observed narrative reports of “notification-driven irritability,” these should be viewed as associations consistent with an observational design rather than direct consequences of platform architecture or “addictive” reinforcement schedules [2, 4].

### Cultural dynamics and digital prioritization

Theoretical explanations involving collectivist cultural dynamics or specific digital design features must be treated as speculative frameworks for future inquiry. While collectivist values may influence how digital connectivity is prioritized—often blurring the lines between social, professional, and personal spheres—the prevalence and impact of social media addiction vary significantly across nations based on diverse cultural values and classification schemes [7].

The interplay between individual trait mindfulness and cultural expectations of “constant availability” may contribute to the observed attention fragmentation [6]. Nevertheless, this study did not directly test the impact of cultural values on digital behavior, and thus these observations remain descriptive rather than explanatory. Recent literature suggests that culturally embedded digital mental health ecosystems may offer scalable and context-sensitive approaches to improving psychological well-being, particularly in rapidly digitizing societies such as India [17].

### Synthesis of behavioral cycles: sleep and nutrition

The qualitative findings regarding the interdependence of digital use, sleep, and nutrition suggest a repetitive behavioral cycle that influences daily mental health resilience. This “Digital-Sleep-Nutrition Resilience Path” aligns with research suggesting that late-night screen exposure disrupts circadian rhythms, which in turn correlates with poor nutritional choices and heightened emotional vulnerability [1, 6, 11, 17].

Addressing attention fragmentation may serve as a critical entry point for broader behavioral interventions aimed at improving sleep hygiene and nutritional stability among young adults [11]. This holistic view of lifestyle risk factors is essential for public health interventions in India, where non-communicable diseases (NCDs) are increasingly linked to digital-age lifestyle shifts.

### Methodological and conceptual scope

The study’s reliance on self-report measures for digital use, the MAAS, and the DASS-21 necessitates an acknowledgment of potential recall and social desirability biases. Furthermore, the operationalization of attention fragmentation via the reverse-scored MAAS represents a conceptual proxy rather than a direct objective measure [8, 9]. While low trait mindfulness is a recognized indicator of attention lapses in high-stimulus environments [2, 6], it may not capture the full multidimensionality of digital fragmentation.

Generalizability is focused on active users, as defined by the inclusion criteria of a minimum of 1.5 hours of daily use. The exclusion of participants with pre-existing psychiatric diagnoses was a deliberate choice to reduce confounding variables, though it limits the application of these findings to clinical populations. Ultimately, these results offer a descriptive snapshot of digital behavior in India, highlighting the need for longitudinal research to untangle the temporal order of these variables [1, 8, 9].

### Limitations and future research

While this study offers preliminary insights into the digital behaviors of Indian adults, several methodological constraints must be considered. First, the reliance on self-report instruments for assessing social media engagement, attention fragmentation, and emotional distress introduces potential reporting and recall biases [6, 15]. Such subjective measures may not always align with objective behavioral data, potentially affecting the precision of the observed associations [11, 15]. Furthermore, the operationalization of attention fragmentation through the reverse-scored MAAS represents a conceptual limitation [2, 6]. While low trait mindfulness is an established indicator of attentional lapses in high-stimulus environments, it may not encompass the full multidimensionality of digital-induced fragmentation without supplementary neuropsychological testing [2, 15].

The generalizability of these findings is also restricted by the specific inclusion and exclusion criteria employed. By requiring a minimum of 1.5 hours of daily social media use, the study focused exclusively on active users, thereby overlooking the nuances of “light” digital engagement [4, 7]. Additionally, the exclusion of individuals with pre-existing psychiatric diagnoses was necessary to mitigate confounding variables, yet this means the results may not be applicable to clinical populations where the interplay between digital habits and distress may manifest differently [3, 5].

Future research should prioritize longitudinal designs to untangle the bidirectional and cross-lagged relationships between digital use and psychological well-being [1]. Incorporating objective tracking data for screen time and physiological markers of stress would provide a more robust validation of the current findings [15]. Moreover, subsequent studies should explore the “Digital-Sleep-Nutrition Resilience Path” using multi-method approaches to better characterize how lifestyle risk factors cluster within the Indian socioeconomic landscape [1, 10].

## Conclusion

This study identifies a significant association between fragmented digital engagement and emotional distress among adults in India, highlighting attention fragmentation as a key correlate in this relationship [1, 2]. The findings suggest that the cumulative impact of frequent digital interruptions—the “social media bytes” phenomenon—is linked to everyday cognitive interference and heightened psychological vulnerability [6]. However, given the observational nature of this work, these results should be interpreted as associations rather than causal links [1, 13].

To promote digital well-being, platform-level interventions such as “focus modes” and public health strategies emphasizing sleep hygiene and nutritional stability are recommended [4, 11]. As India continues its rapid digital transition, addressing the cognitive and behavioral impacts of constant connectivity will be essential for mitigating the rising burden of lifestyle-related mental health challenges [1, 7].

## Supplementary Information

Supplementary Material 1.

## Data Availability

Anonymized transcripts are available upon reasonable request, subject to ethical approval from the Institutional Ethics Committee of Datta Meghe Institute of Higher Education and Research.
