# Supplementary material for: Attention fragmentation and emotional distress: a mixed-methods study of social media use among Indian adults aged 18–45 years
Source: BMC Psychol. 2026 May 26;14:1080. doi: 10.1186/s40359-026-04846-2 (PMC13390226; doi:10.1186/s40359-026-04846-2)
Supplement: Supplementary file 1 — Supplementary Material 1. [file 40359_2026_4846_MOESM1_ESM.docx]

**Supplementary File 1**

**Full Semi-Structured Interview Guide**

**Attention Fragmentation and Emotional Distress: A Mixed-Methods Study of Social Media Use Among Indian Adults Aged 18–45 Years**

Bayaskar S & Sharma D

**Interview Guide Overview**

This semi-structured guide was used for face-to-face interviews (45–60 minutes) with 32 purposively selected participants (16 high-fragmentation, 16 moderate-fragmentation based on MAAS tertiles). The guide was developed iteratively during pilot testing (n = 12) and refined for cultural appropriateness and clarity in English, Hindi, and Marathi.

All interviews began with rapport-building, explanation of the study purpose, voluntary participation, confidentiality, and right to withdraw. Audio recording was initiated only after explicit consent. Probes were used flexibly to elicit concrete examples and deeper emotional reflections. Field notes captured non-verbal cues and contextual observations.

**Opening / Rapport-Building (5–7 minutes)**

1. Thank you for agreeing to speak with me today. Could you briefly tell me a little about yourself — your age group, what you do (studies/work), and where you’re from? *(Purpose: Build comfort, confirm eligibility, establish context)*
2. How would you describe your typical day in terms of social media use — when do you usually start checking, which apps/platforms do you use most? *(Purpose: Ease into the topic, obtain baseline usage narrative)*

**Core Questions & Probes (30–40 minutes)**

1. Can you walk me through a recent typical session of using social media?
   - How often do you find yourself switching between apps or getting pulled away from one thing to another?
   - Probe: Give me an example from the last day or two — what happened step by step?
   - Probe: How many times in that session did you feel your attention shift unexpectedly?
2. What does it feel like in your mind or body when your focus keeps getting interrupted by notifications, new posts, messages, or videos?
   - Probe: Can you describe any physical sensations (e.g., tension, restlessness) or mental feelings (e.g., scattered, overwhelmed)?
   - Probe: Does it feel different depending on the platform (e.g., Reels vs. WhatsApp vs. YouTube Shorts)?
3. Which types of content or apps most often cause your attention to break away from what you were doing? Why do you think that happens?
   - Probe: Is there a difference between passive scrolling (e.g., watching Reels) and active responding (e.g., replying to messages or status updates)?
   - Probe: How do notifications from family groups, friends, or work affect this?
4. How do these attention interruptions affect your emotions or mood — for example, do they make you feel anxious, irritated, low, guilty, or something else?
   - Probe: Can you describe a recent time when an interruption led to feeling emotionally drained, upset, or overwhelmed?
   - Probe: Do you notice any changes in how positive or motivated you feel after a fragmented session?
5. In what ways do family, friends, college/work expectations, or social norms influence how much you stay connected or respond quickly?
   - Probe: How do these social factors make it harder or easier to maintain focus?
   - Probe: Have you ever felt pressure to reply immediately even when you were trying to concentrate on something else?
6. Have you tried any ways to reduce these attention interruptions or manage the emotional effects?
   - Probe: What strategies have you used (e.g., turning off notifications, setting time limits, using focus modes)?
   - Probe: What worked well, what didn’t, and why?
   - Probe: What would help you feel more in control of your attention and mood when using social media?

**Closing (5–7 minutes)**

1. Is there anything else you would like to share about how social media affects your attention or emotions that we haven’t covered? *(Purpose: Capture any additional insights or missed perspectives)*
2. How are you feeling after talking about this? Do you have any questions for me? *(Purpose: Check for distress, offer referral if needed, thank participant)*
